# Estrogen Metabolism-Associated CYP2D6 and IL6-174G/C Polymorphisms in *Schistosoma haematobium* Infection

**DOI:** 10.3390/ijms18122560

**Published:** 2017-11-28

**Authors:** Rita Cardoso, Pedro C. Lacerda, Paulo P. Costa, Ana Machado, André Carvalho, Adriano Bordalo, Ruben Fernandes, Raquel Soares, Joachim Richter, Helena Alves, Monica C. Botelho

**Affiliations:** 1 Department of Health Promotion and Chronic Diseases, National Institute of Health Dr. Ricardo Jorge (INSA), Rua Alexandre Herculano 321, 4000-055 Porto, Portugal; rita.cardoso@insa.min-saude.pt (R.C.); m.helena.alves@insa.min-saude.pt (H.A.); 2 Department of Human Genetics, National Institute of Health Dr. Ricardo Jorge (INSA), Rua Alexandre Herculano 321, 4000-055 Porto, Portugal; pedro.lacerda@insa.min-saude.pt (P.C.L.); Paulo.costa@insa.min-saude.pt (P.P.C.); 3 Instituto de Ciências Biomédicas Abel Salazar (ICBAS/UP), Universidade do Porto, Rua Jorge Viterbo Ferreira 228, P 4050-313 Porto, Portugal; ammachado@icbas.up.pt (A.M.); bordalo@icbas.up.pt (A.B.); 4 Centro Interdisciplinar de Investigação Marinha e Ambiental (CIIMAR/CIMAR), Universidade do Porto, Av. General Norton de Matos s/n, 4450-208 Matosinhos, Portugal; 5 Division of Endocrinology, Diabetes and Metabolism, Santo Antonio Hospital—Centro Hospitalar do Porto (CHP), Largo do Prof. Abel Salazar, 4099-001 Porto, Portugal; andre.carvalho@chporto.min-saude.pt; 6 Escola Superior de Saúde, Instituto Politécnico do Porto, Rua Dr. António Bernardino de Almeida, 400, 4200-079 Porto, Portugal; ruben@ess.ipp.pt; 7 Unit of Metabolism, Nutrition and Endocrinology, Instituto de Investigação e Inovação da Universidade do Porto (i3S), Rua Alfredo Allen, 4200-135, Porto, Portugal; raqsoa@med.up.pt; 8 Departamento de Biomedicina, Unidade de Bioquímica, Faculdade de Medicina, Universidade do Porto, Al. Prof. Hernâni Monteiro, 4200-319 Porto, Portugal; 9 Institute of Tropical Medicine and International Health, Charité—Universitätsmedizin Berlin, Augustenburger Platz 1, 13353 Berlin, Germany; joachim.richter@charite.de; 10 Fundação Professor Ernesto Morais, Rua de Monsanto 512, 4250-288 Porto, Portugal

**Keywords:** estrogen biosynthesis, estrogen metabolism, BMI, *S. haematobium*-associated bladder cancer

## Abstract

*Schistosoma haematobium* is a human blood fluke causing a chronic infection called urogenital schistosomiasis. Squamous cell carcinoma of the urinary bladder (SCC) constitutes chronic sequelae of this infection, and *S. haematobium* infection is accounted as a risk factor for this type of cancer. This infection is considered a neglected tropical disease and is endemic in numerous countries in Africa and the Middle East. Schistosome eggs produce catechol-estrogens. These estrogenic molecules are metabolized to active quinones that induce modifications in DNA. The cytochrome P450 (CYP) enzymes are a superfamily of mono-oxygenases involved in estrogen biosynthesis and metabolism, the generation of DNA damaging procarcinogens, and the response to anti-estrogen therapies. IL6 Interleukin-6 (IL-6) is a pleiotropic cytokine expressed in various tissues. This cytokine is largely expressed in the female urogenital tract as well as reproductive organs. Very high or very low levels of IL-6 are associated with estrogen metabolism imbalance. In the present study, we investigated the polymorphic variants in the *CYP2D6* gene and the C-174G promoter polymorphism of the *IL-6* gene on *S. haematobium*-infected children patients from Guine Bissau. *CYP2D6* inactivated alleles (28.5%) and *IL6*G-174C (13.3%) variants were frequent in *S. haematobium*-infected patients when compared to previously studied healthy populations (4.5% and 0.05%, respectively). Here we discuss our recent findings on these polymorphisms and whether they can be predictive markers of schistosome infection and/or represent potential biomarkers for urogenital schistosomiasis associated bladder cancer and infertility.

## 1. Introduction

*Schistosoma haematobium* is a human blood fluke causing a chronic infection called urogenital schistosomiasis. Squamous cell carcinoma of the urinary bladder (SCC) constitutes chronic sequelae of this infection, and *S. haematobium* infection is accounted as a risk factor for this type of cancer. This infection is considered a neglected tropical disease and is endemic in many countries of Africa and the Middle East [1].

*S. haematobium* is endemic in 53 countries in the Middle East and in most of the African continent, including the islands of Madagascar and Mauritius. Following successful eradication programs, the infection is no longer of public health importance in Egypt, Lebanon, Oman, Syria, Tunisia and Turkey since transmission is low or nonexistent. A borderline and indefinable focus is still in existence in India and requires additional evidence [2].

Infection with *Schistosoma* spp. affects more than 258 million people worldwide. Praziquantel is the main antihelminthic drug currently used to treat this infection. This drug is effective in eliminating adult worms, but is unsuccessful in the prevention of re-infection and does not treat severe liver damage nor bladder cancer [3].

Schistosome eggs produce catechol-estrogens. These estrogenic molecules are metabolized by cytochrome P450 oxygenases to active quinones that cause alterations in DNA, known to promote breast or thyroid cancer [4,5,6]. Our group has shown that schistosome egg-associated catechol estrogens induce tumor-like phenotypes in urothelial cells, possibly due to the formation of parasite estrogen-host cell chromosomal DNA adducts [5]. These estrogen metabolites also contribute to schistosomiasis-associated infertility [6].

The cytochrome P450 (*CYP*) supergene family encompasses a cluster of oxygenases that play a key role in the metabolism of a miscellaneous group of endogenous substrates such as fatty acids, steroids, and vitamin D as well as exogenous compounds including phytochemicals, environmental pollutants, and pharmaceuticals [7]. Given the vital function of *CYP* genes in the biosynthesis of steroids, especially estrogen, altered expression of *CYP*s might contribute to the development and proliferation of tumor cells and increase tumor growth through the activation of procarcinogens. Specifically *CYP2D6* encodes a critical enzyme on estrogen biosynthesis and metabolism, and the outcome of this *CYP* gene variant can have a downstream cost on patient response [8].

Interleukin-6 (IL-6) is a pleiotropic proinflammatory cytokine, vastly expressed in the female urogenital tract and reproductive organs. It has been given a role in estrogen metabolism imbalance. The promoter region of the *IL-6* gene is dynamically regulated at multiple sites, as well as the 23 base-pair “multiple response element” site, which is activated by interleukin-1, tumor necrosis factor alpha, and other factors [9]. The C-174G promoter polymorphism of the *IL-6* gene has been established to control transcriptional regulation [10] and has been associated with plasma IL-6 levels in patients with systemic-onset juvenile chronic arthritis and in patients with primary Sjögren’s syndrome [11].

To the best of our knowledge, despite the established role of *CYP2D6* and *IL6* in estrogen metabolism, there are no studies addressing these gene variants in *S. haematobium*-infected patients. In the present study, we investigated polymorphic variants in *CYP2D6* and the -174 G/C (rs1800795) promoter polymorphism of the *IL-6* gene on a cohort of *S. haematobium*-infected children from Guinea-Bissau.

## 2. Results

### 2.1. CYP 2D6 Alleles *3, *4 and *5/*5 in S. haematobium-Infected Patients

**Figure 1:**
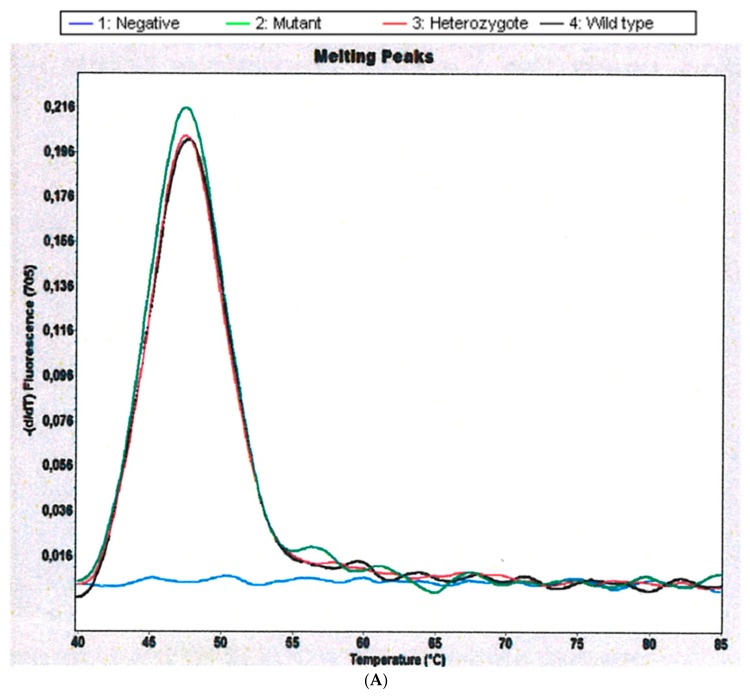

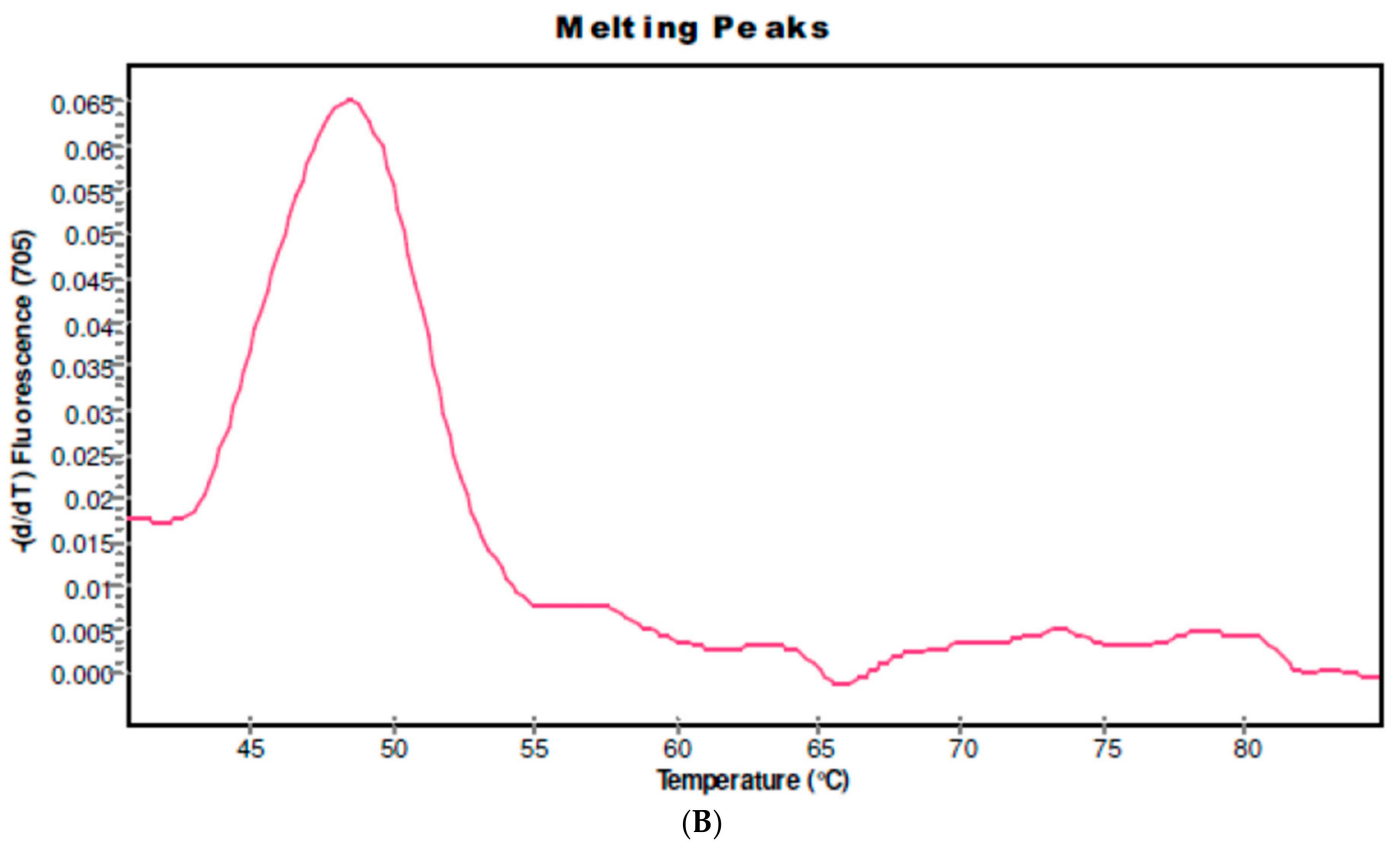
Genotyping of CYP2D6 *3*4*5/*5*. (**A**) Derivative melting curve plots—dF/dT vs. temperature. **Red** = Wildtype. **Black** = Heterozygote. **Green** = Mutant. (**B**) Genotyping in a mutant patient. In the case where no melting signals are visible, but the control gene has been amplified as shown by the melting point at 48.0 °C, the *CYP 2D6* gene is deleted (*5/*5).

From the 18 patients studied, we obtained frequencies of *CYP2D6* for 14 patients. Mutant samples were analyzed in Channel 705 of Light Cycler 2.0 Instrument showing melting peaks for *hCR5* (amplification control) at 47.5 °C. The *CYP 2D6* Alleles *3 and *4 were not found in any of the samples studied. In contrast, we found that 4 of 14 (28.5%) schistosomiasis-haematobia-infected patients are carriers of the inactivated allele CYP2D6*5, which is characterized by a deletion of the entire *CYP2D6* gene (Table 1 and Figure 1). Microhaematuria was found in all of the *CYP2D6* inactivated allele carriers and only in 80% of non-carriers. Age and body mass index (BMI) were not significantly different between the two groups.

### 2.2. IL6-174C Variant in S. haematobium Infected Patients

**Figure 2:**
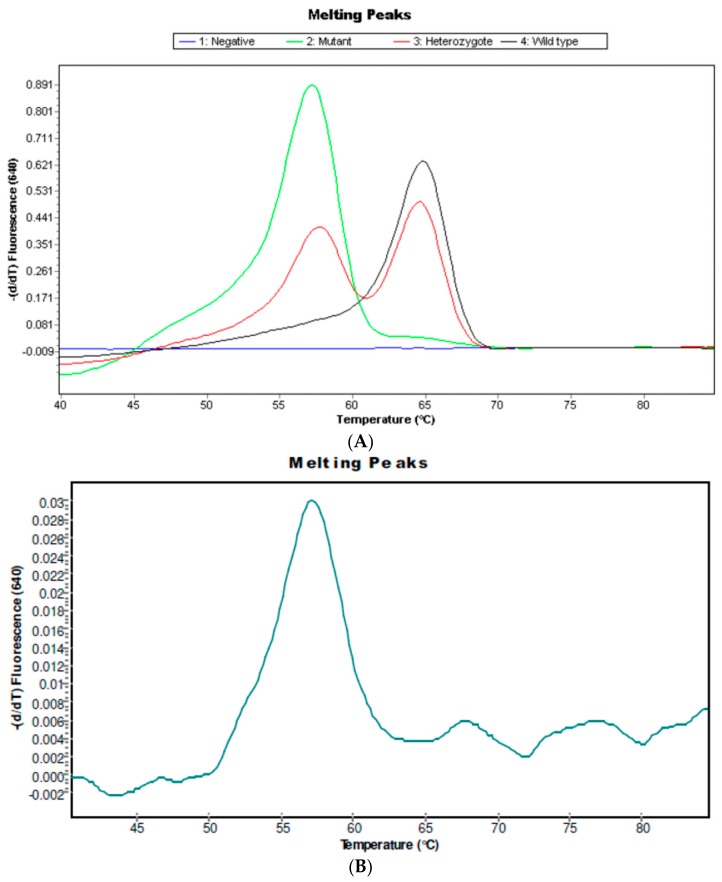
Genotyping of the promoter *IL6 G-174C* region. (**A**) Derivative melting curve plots—dF/dT vs. temperature. **Red** = Wildtype. **Black** = Heterozygote. **Green** = Mutant. (**B**) Genotyping in a mutant patient. The presence of a melting peak at 57 °C indicate a mutant patient corresponding with *IL6-174C7C* genotype.

Fifteen out of the 18 patients studied presented the *IL6-174C* variant. Mutant samples were analyzed in Channel 640 of the LightCycler 2.0 Instrument showing melting peaks at 57 °C. The *IL6-174C* variant was found in 2 of 15 (13.3%) schistosomiasis-haematobia-infected patients (Table 2 and Figure 2). The two patients carrying the mutant genotype were younger than the ones with the wild type (WT) (6.5 ± 0.7 vs. 10.1 ± 3.1; *p* = 0.005) and presented a lower BMI (10.6 ± 5.9 vs. 14.8 ± 1.8; *p* = 0.04). Microhaematuria was present in one of the mutant carriers (50%) and in 9 of the 13 (69%) WT carrier patients.

## 3. Discussion

This preliminary study with a limited number of patients suggested that CYP2D6 *5/*5 and *IL6-174C* polymorphisms has an effect on severity and morbidity of schistosomiasis. In the present study, we found that 28.5% of schistosomiasis-infected patients were carriers of CYP2D6 *5/*5. To our knowledge, this is the first study of this polymorphism conducted on an African population. According to [12], the frequency of this allele in a healthy population is 4.5%. The cytochrome P450 enzyme debrisoquine 4-hydroxylase (CYP2D6) metabolizes countless diverse classes of universally used drugs and toxins. Due to autosomal recessive inheritance of two mutant *CYP2D6* null alleles, individuals are classified as carriers of the inactivated allele CYP2D6*5, which is characterized by a deletion of the entire *CYP2D6* gene and confers the phenotype of poor metabolizers (PM) [13]. Poor metabolizer subjects might acquire toxic plasma concentrations and adverse drug reactions. Additionally, mutant *CYP2D6* alleles have been implicated as a predictor of susceptibility for diseases such as cancer and for neurological disorders [13]. In our study, 28.5% are classified as poor metabolizers. Given the fact that in the present study 4 of 5 (80%) of the poor metabolizers are underweight (BMI < 15), a feature associated in our previous study with *S. haematobium* infection [14], it is likely that this genotype might increase the susceptibility to infection and morbidity of this parasite [13].

Our group has been involved in the identification of parasite-derived substances that might be implicated in the host–parasite interactions of schistosomes [15]. The bulk of these substances are catechol estrogens. The genotoxic effects of these estrogen metabolites are ascribed to oxidation of catechol estrogens to quinones, followed by redox cycling and the formation of reactive oxygen species that sequentially react with DNA [16]. These electrophilic compounds are able to react with DNA to form depurinating adducts [17]. It is conceivable that apurinic sites in chromosomal DNA that result from this reaction generate mutations that might underlie the carcinogenic effect of schistosomes [1,18]. Given the context of the unarguable link between imbalance in the metabolism of estrogens and the production of depurinating estrogen–DNA adducts, the presence of schistosomiasis-derived estrogen metabolites may have practical consequences in the growth development of infected children carriers of the CYP2D6 *5/*5 allele.

We observed that 174 G/C (rs1800795) promoter polymorphism of the *IL-6* gene was found in 2 of 15 (13.3%) of *S. haematobium*-infected individuals. The frequency of this variant in a healthy African population is 0.05% [10]. These authors also studied Caucasians and Gujarati Indians and found a frequency of 0.4% and 0.1%, respectively, for the mutant *IL6-174 C*/*C* genotype [10]. The presence of the mutant genotype results in a lower IL-6 expression after a given inflammatory stimulus compared with the wild-type genotype [10]. Therefore, the presence of the mutant genotype in *S. haematobium*-infected patients therefore suggests that this genotype confers a susceptibility influence for the development of the disease. We also found it to be significantly associated with lower BMI (10.6 ± 5.9 vs. 14.8 ± 1.8; *p* = 0.04), indicating that infected carriers of this variant might have an increased risk of developing schistosomiasis-associated chronic sequelae at a much younger age. Concerning the role of IL6 in estrogen metabolism, there is emerging evidence linking IL6 deficiency with reproductive impairment, leading to in how this cytokine contributes to infertility [19]. This is in accordance with our recent new findings that schistosomiasis is associated with infertility and suboptimal fecundity [6].

Altogether, the current survey provides primary data on the frequency of inactivating alleles of *CYP2D6* and *IL6* G-174C polymorphisms in *S. haematobium*-infected patients. Despite a limited number of patients, we found an appalling increase in the frequencies of *CYP2D6* *5/*5 and *IL6*-174C/C genotypes in comparison to previously studied healthy populations, including populations of healthy African subjects previously studied. The presence of these genotypes could explain schistosomiasis-associated cancer and infertility and may represent potential predictors for growth development and metabolism disorders in these patients. On the other hand, they may have prognostic significance, namely, regarding the development of cancer and infertility, something that will need to be addressed in further studies.

## 4. Material and Methods

### 4.1. Study Area, Population and Design

This research (PTDC/AAC-CLI/103539/2008) was carried out in compliance with the Helsinki Declaration and with the approval of the Executive Board of the Institute of Biomedical Sciences Abel Salazar of Porto University.

The study was conducted in a children population from Guinea-Bissau (West Africa) in early September 2011 during the peak of the wet season.

Eighteen schoolchildren aged 6–13 infected with *S. haematobium* were targeted in this study. The purpose of the study was explained to all childrens’ parents, and individual informed consent was obtained.

### 4.2. Urine Collection

Following the anthropometric measurements, each child was asked to urinate in a plastic cup. Urine (50–200 mL) was immediately transferred to 15 mL sterile non-heparinized vacuum tubes, and kept refrigerated in cool boxes.

### 4.3. Urine Analysis

Upon collection, urine was checked for microhaematuria by means of appropriate reagent strips (Combur^®^. Roche Diagnostics Division, Basel, Switzerland). In the laboratory, in Portugal, the presence of eggs of *S. haematobium* was detected and quantified by microfiltration of 10 mL of urine through nucleopore filters [20].

### 4.4. Anthropometric Measurements

Body weight and height were measured using a standardized method of anthropometric techniques (WHO, 1995). Height was measured to the nearest 0.1 cm, and weight was measured to the nearest 0.1 kg using portable digital scales. Body mass index (BMI) of each child was calculated. BMI < 15 kg/m^2^ was considered underweight [21].

### 4.5. DNA Collection and Extraction

Genomic DNA was extracted from urine sediments using High Pure PCR Template Preparation kits (Roche Diagnostics, GmbH, Mannheim, Germany) [22].

### 4.6. Genetic Analysis

#### 4.6.1. Detection of CYP 2d6 Alleles *3, *4 and *5/*5

We used Lightmix Kit for the detection of CYP 2D6 Alleles *3, *4, and *5/*5. This kit provides a fast, easy, and accurate system to identify CYP 2D6 Alleles *3 and *4 as well as a homozygous deletion of the gene (*5/*5) in a nucleic acid extract according to the manufacturer (TIBMolBiol GmbH, Berlin, Germany) [23]. After amplification with specific primers, the genotypes were identified through specific melting points (Tm) recorded during the melting curve analysis. For identification of Allele *3, a 317 bp fragment from Exon 5 was amplified and analyzed with a SimpleProbe oligomer (Channel 530) depicting a Tm of 60.2 °C for the wild-type allele and 55.0 °C for the deletion 2637delA allele. For analysis of Allele *4, a 336 bp fragment spanning the Intron 3–Exon 4 junction was generated and analyzed with LightCycler Red 640 labeled hybridization probes, exhibiting a Tm of 56.3 °C for the wild-type allele and 64.5 °C for the variant 1934A. The deletion of the entire *CYP2D6* gene (CYP 2D6*5/*5) did not produce any signal in Channels 530 or 640. In this case, to demonstrate the presence of amplifiable DNA in these biological samples, a 234 bp fragment of the human chemokine receptor type 5 (hCR5) was co-amplified with specific primers. The hCR5 amplification was detected using hybridization probes labeled with LightCycler Red 690 (Channel 705), exhibiting a specific melting peak at a Tm of 48 °C.

#### 4.6.2. Detection of IL6 G-174C

We used Lightmix Kit for the detection of IL6 G-174C. This kit provides a fast, easy, and accurate system for identifying the genotype of IL6 G-174C in a nucleic acid extract according to the manufacturer (TIBMolBiol, GmbH, Berlin, Germany) [24]. A 175 bp fragment of the human *IL6* gene spanning the promoter IL6 G-174C region was amplified with specific primers. The resulting PCR fragments were analyzed with hybridization probes labeled with LightCycler Red 640. The genotype was identified by running a melting curve with specific melting points (Tm). The wild-type allele IL6 G-174C exhibited a Tm of 64.0 °C in Channel 640. The allele variant IL6-174C exhibited a Tm of 57.0 °C in Channel 640.

#### 4.6.3. PCR Experiment Protocol

A total of 20 μL of PCR mixture containing 2–5 μL of sample DNA according to Roche’s datasheet of LightCycler FastStart DNA Master Plus HybProbe (Roche Diagnostics, Mannheim, Germany) was used [25]. The LC PCR assay was performed on the LightCycler 2.0 Instrument (Roche Diagnostics, Mannheim, Germany) with an initial denaturation at 95 °C for 10 min, followed by 45 cycles with denaturation at 95 °C for 5 s, 60 °C for 10 s, and 72 °C for 15 s. After amplification cycles, the reaction mixture was denatured at 95 °C for 20 s, held at 40 °C for 20 s followed by one step at 40 °C for 30 s, and gradually heated to 85 °C at a rate of 0.2 °C/s. The melting curves were converted to melting peaks by plotting the negative derivative of the fluorescent signal with respect to temperature [d(F2)/dT]. In this way, the presence of a mutant heteroduplex (containing the wild-type sequences and the mutant allele) is easily detectable because of its low melting temperatures.

### 4.7. Statistical Analysis

For the group comparison, chi-square tests with Yate’s correction were used or with Fisher’s exact test (two-sided) when expected values were below 5. For independent samples, a Student’s *t*-test was used for the comparison of means (OpenEpi software, version 3.03, Atlanta, GA, USA).

## 5. Conclusions

Altogether, the current survey provides primary data on the frequency of inactivating alleles of *CYP2D6* and *IL6* G-174C polymorphisms in *S. haematobium*-infected patients. Despite a limited number of patients, we found an appalling increase in the frequencies of *CYP2D6* *5/*5 and *IL6*-174C/C genotypes in comparison to previously studied healthy populations, including those of African subjects. The presence of these genotypes could explain schistosomiasis-associated cancer and infertility and may represent potential predictors for growth development and metabolism disorders in these patients. On the other hand, they may have prognostic significance, namely regarding the development of cancer and infertility, something that will need to be addressed in further studies.

## Figures and Tables

**Table 1:** Population characteristics of CYP2D6*5/*5 carriers vs. wild type in children infected with *S. haematobium*.

Population Characteristics	*CYP*; *n* = 4 (28.5%)	WT; *n* = 10 (71.4%)	*p* Value	OR	95% CI
Age (years, median ± SD)	10.75	10.8	n.s.		
Female	1	4	n.s.	0.5242	0.01537, 7.015
Male	3	6	n.s.	1.908	0.1426, 65.05
Microhaematuria (%)	4	8	n.s.	1.599	0.07674, 72.45
BMI (median ± SD)	15.2	15.5	n.s.		

CYP—genotype CYP2D6*5/*5; CI—confidence interval; SD—standard deviation; BMI—body mass index; OR—odds ratio; n.s.—not significant; WT—wild type.

**Table 2:** Population characteristics of *IL6-174C*/*C* carriers vs. wild type in children infected with *S. haematobium*.

Population Characteristics	IL6; *n* = 4 (28.5%)	WT; *n* = 10 (71.4%)	*p* Value	OR	95% CI
Age (years, median ± SD)	6.5 ± 0.7	10.1 ± 3.1	0.005		
Female	1	5	n.s.	1.549	0.03441, 69.74
Male	1	8	n.s	0.6455	0.01434, 29.06
Microhaematuria (%)	1	9	n.s	0.4714	0.01033, 21.51
BMI (median ± SD)	10.6 ± 5.9	14.8 ± 1.8	0.04		

IL6—genotype *IL6-174C*/*C*; CI—confidence interval; SD—standard deviation; BMI—body mass index; OR—odds ratio; n.s.—not significant; WT—wild type.

## Abbreviations

SCC	Squamous Cell Carcinoma
CYP	Cytochrome P450
IL-6	Interleukin 6
BMI	Body Mass Index
WT	Wild-Type
PM	Poor Metabolizer

## References

[1] Botelho M.C., Alves H., Barros A., Rinaldi G., Brindley P.J., Sousa M. (2015). The role of estrogens and estrogen receptor signaling pathways in cancer and infertility: The case of schistosomes. Trends Parasitol..

[2] Botelho M.C., Machado J.C., Brindley P.J., Correia da Costa J.M. (2011). Targeting molecular signaling pathways of *Schistosoma haemotobium* infection in bladder cancer. Virulence.

[3] Koslowski N., Sombetzki M., Loebermann M., Engelmann R., Grabow N., Österreicher C.H., Trauner M., Mueller-Hilke B., Reisinger E.C. (2017). Single-sex infection with female *Schistosoma mansoni* cercariae mitigates hepatic fibrosis after secondary infection. PLoS Negl. Trop. Dis..

[4] Botelho M.C., Soares R., Vale N., Ribeiro R., Camilo V., Almeida R., Medeiros R., Gomes P., Machado J.C., Correia da Costa J.M. (2010). *Schistosoma haematobium*: Identification of new estrogenic molecules with estradiol antagonistic activity and ability to inactivate estrogen receptor in mammalian cells. Exp. Parasitol..

[5] Botelho M.C., Vale N., Gouveia M.J., Rinaldi G., Santos J., Santos L.L., Gomes P., Brindley P.J., Correia da Costa J.M. (2013). Tumour-like phenotypes in urothelial cells after exposure to antigens from eggs of *Schistosoma haematobium*: An oestrogen-DNA adducts mediated pathway?. Int. J. Parasitol..

[6] Santos J., Gouveia M.J., Vale N., Delgado Mde L., Gonçalves A., da Silva J.M., Oliveira C., Xavier P., Gomes P., Santos L.L. (2014). Urinary estrogen metabolites and self-reported infertility in women infected with *Schistosoma haematobium*. PLoS ONE.

[7] Nebert D.W., Russell D.W. (2002). Clinical importance of the cytochromes P450. Lancet.

[8] Blackburn H.L., Ellsworth D.L., Shriver C.D., Ellsworth R.E. (2015). Role of cytochrome *P450* genes in breast cancer etiology and treatment: Effects on estrogen biosynthesis, metabolism, and response to endocrine therapy. Cancer Causes Control.

[9] Terry C.F., Loukaci V., Green F.R. (2000). Cooperative influence of genetic polymorphisms on interleukin 6 transcriptional regulation. J. Biol. Chem..

[10] Fishman D., Faulds G., Jeffery R., Mohamed-Ali V., Yudkin J.S., Humphries S., Woo P. (1998). The effect of novel polymorphisms in the interleukin-6 (*IL-6*) gene on IL-6 transcription and plasma IL-6 levels, and an association with systemic-onset juvenile chronic arthritis. J. Clin. Investig..

[11] Hulkkonen J., Pertovaara M., Antonen J., Pasternack A., Hurme M. (2001). Elevated interleukin-6 plasma levels are regulated by the promoter region polymorphism of the *IL6* gene in primary Sjogren’s syndrome and correlate with the clinical manifestations of the disease. Rheumatology.

[12] Gaedigk A., Blum M., Gaedigk R., Eichelbaum M., Meyer U.A. (1991). Deletion of the entire cytochrome P450 *CYP2D6* gene as a cause of impaired drug metabolism in poor metabolizers of the debrisoquine/sparteine polymorphism. Am. J. Hum. Genet..

[13] Steen V.M., Molven A., Aarskog N.K., Gulbrandsen A.K. (1995). Homologous unequal cross-over involving a 2.8 kb direct repeat as a mechanism for the generation of allelic variants of human cytochrome P450 *CYP2D6* gene. Hum. Mol. Genet..

[14] Botelho M.C., Machado A., Carvalho A., Vilaça M., Conceição O., Rosa F., Alves H., Richter J., Bordalo A.A. (2016). *Schistosoma haematobium* in Guinea-Bissau: Unacknowledged morbidity due to a particularly neglected parasite in a particularly neglected country. Parasitol. Res..

[15] Botelho M.C., Ribeiro R., Vale N., Oliveira P., Medeiros R., Lopes C., Machado J.C., Correia da Costa J.M. (2012). Inactivation of estrogen receptor by *Schistosoma haematobium* total antigen in bladder urothelial cells. Oncol. Rep..

[16] Fussell K.C., Udasin R.G., Smith P.J., Gallo M.A., Laskin J.D. (2011). Catechol metabolites of endogenous estrogens induce redox cycling and generate reactive oxygen species in breast epithelial cells. Carcinogenesis.

[17] Cavalieri E.L., Rogan E.G. (2016). Depurinating estrogen-DNA adducts, generators of cancer initiation: Their minimization leads to cancer prevention. Clin. Transl. Med..

[18] Botelho M.C., Alves H., Richter J. (2017). Estrogen catechols detection as biomarkers in schistosomiasis induced cancer and infertility. Lett. Drug Des. Discov..

[19] Prins J.R., Gomez-Lopez N., Robertson S.A. (2012). Interleukin-6 in pregnancy and gestational disorders. J. Reprod. Immunol..

[20] Botelho M.C., Sousa M. (2014). New biomarkers to fight urogenital schistosomiasis: A major neglected tropical disease. Biomark. Med..

[21] World Health Organization (1995). Physical Status: The Use and Interpretation of Anthropometry.

[22] Vogelstein B., Gillespie D. (1979). Preparative and analytical purification of DNA from agarose. Proc. Natl. Acad. Sci. USA.

[23] Sistonen J., Sajantila A., Lao O., Corander J., Barbujani G., Fuselli S. (2007). CYP2D6 worldwide genetic variation shows high frequency of altered activity variants and no continental structure. Pharmacogenet. Genom..

[24] Sawczenko A., Azooz O., Paraszczuk J., Idestrom M., Croft N.M., Savage M.O., Ballinger A.B., Sanderson I.R. (2005). Intestinal inflammation-induced growth retardation acts through IL-6 in rats and depends on the -174 IL-6 G/C polymorphism in children. Proc. Natl. Acad. Sci. USA.

[25] Weise A., Prause S., Eidens M., Weber M.M., Kann P.H., Forst T., Pfützner A. (2010). Prevalence of *CYP450* gene variations in patients with type 2 diabetes. Clin. Lab..

